# Mitochondrial Transplantation Alleviates Doxorubicin-Induced Toxicity in Rat Renal Cells

**DOI:** 10.5812/ijpr-146033

**Published:** 2024-03-31

**Authors:** Enayatollah Seydi, Mahsa Andalib, Sana Yaghoubi, Amir Fakhri, Jale Yuzugulen, Abdollah Arjmand, Jalal Pourahmad

**Affiliations:** 1 Department of Occupational Health and Safety Engineering, School of Health, Alborz University of Medical Sciences, Karaj, Iran; 2 Research Center for Health, Safety and Environment, Alborz University of Medical Sciences, Karaj, Iran; 3 School of Pharmacy, Shahid Beheshti University of Medical Sciences, Tehran, Iran; 4 School of Pharmacy, Eastern Mediterranean University, Famagusta, North Cyprus

**Keywords:** Doxorubicin, Oxidative Stress, Nephrotoxicity, Mitochondria Transplantation, Renal Proximal Tubular Cells

## Abstract

**Background:**

Doxorubicin (DOX) is used in the treatment of various cancers and has good effectiveness. However, its therapeutic use is limited due to its effects on various organs and healthy cells. Doxorubicin can affect the kidneys and cause toxicity. Evidence shows that DOX induces nephrotoxicity through oxidative stress.

**Objectives:**

In this research, we examined the effect of mitochondrial transplantation on improving mitochondrial and cellular toxicity caused by DOX on renal proximal tubular cells (RPTCs).

**Methods:**

The research measured 7 toxicity parameters, including cell lysis, reactive oxygen species (ROS) formation, mitochondrial membrane potential (MMP) decline, GSH and GSSG content, lipid peroxidation (LPO), adenosine triphosphate (ATP) content, and Caspase-3 activity (the final mediator of apoptosis). Active fresh mitochondria were prepared from Wistar rat kidney.

**Results:**

The findings indicated that DOX caused cytotoxicity in RPTCs. Additionally, DOX induced oxidative stress by increasing the level of reactive oxygen species, reducing glutathione content, and elevating lipid peroxidation. Moreover, it led to damage to the mitochondrial membrane, increased caspase-3 activity, and decreased ATP content. Mitochondrial transplantation, as a new therapeutic approach, reduced oxidative stress, mitochondrial membrane damage, and apoptosis caused by DOX in RPTCs. Furthermore, this therapeutic approach increased the ATP content in RPTCs.

**Conclusions:**

Our study suggests that this therapeutic approach could be helpful in the treatment of drug-induced nephrotoxicity.

## 1. Background

Drugs are exogenous compounds that can damage various human organs, including the kidneys (1). The kidney is known as a vital and key organ in the body of living organisms and is involved in many essential functions (2, 3). Drugs are one of the causes of kidney toxicity that leads to many disorders in this organ (4, 5). In various studies, the relationship between the use of anticancer drugs and kidney toxicity has been reported (6, 7). Doxorubicin (DOX) is one of the anti-cancer drugs that is effective in treating various human cancers (8, 9). Despite the good effectiveness of DOX in the treatment of several cancers, due to its toxic effects on some organs such as the kidneys, its therapeutic use is limited. Also, DOX can target healthy cells and cause side effects in these cells (10-12). In the kidneys, proximal tubules are one of the most important targets of nephrotoxic drugs (13).

The mechanism of nephrotoxicity caused by DOX is not well understood. Nevertheless, evidence shows that DOX causes nephrotoxicity through the production of reactive oxygen species (ROS) and induction of oxidative stress (14, 15). The consequence of an imbalance between the level of oxidants (ROS) and antioxidants is oxidative stress, in which oxidant agents can cause serious damage to cells (16). Mitochondria are considered the main sources of ROS production in living organisms. The production of ROS often occurs due to disturbances in the mitochondrial respiratory chain (17, 18). Doxorubicin can affect the mitochondrial transmission chain and mitochondrial pathways, which can cause the production of ROS, dysfunction of mitochondria, and apoptosis (19, 20). Also, it has been reported that DOX can induce apoptosis in proximal tubule cells by stimulating the mitochondrial pathway and activating caspase-3 (21, 22).

Mitochondria are known as one of the vital organelles in cells. These organelles are involved in many physiological processes (20). Mitochondria are involved in the production of ROS, adenosine triphosphate (ATP), and induction of death signaling. Normal levels of ROS are involved in many physiological processes. In abnormal conditions, they cause damage to vital macromolecules in the cell (23, 24). Dysfunction of mitochondria is involved in the pathogenesis of many diseases (especially mitochondrial diseases) and also cell viability (25, 26). Therefore, recovery of dysfunctional mitochondria is of great importance. Mitochondria transplantation is one of the new therapeutic approaches to improve mitochondrial diseases. This approach is based on the transfer of exogenous normal mitochondria to dysfunctional cells or tissues to improve mitochondrial function (27, 28).

## 2. Objectives

The novelty of this study lies in examining the protective effects of mitochondrial transplantation on cytotoxicity and oxidative stress through the measurement of several parameters such as ROS generation, reduced glutathione (GSH) and oxidized glutathione (GSSG) content, changes in mitochondrial membrane, apoptosis by assessing caspase-3 activity, and ATP content caused by the treatment of rat renal cells with DOX. These parameters have not been thoroughly investigated in other studies. Due to the lack of sufficient information related to DOX nephrotoxicity, this study aimed to investigate the effects of mitochondrial transplantation on DOX-induced nephrotoxicity.

## 3. Methods

### 3.1. Animals

The animals (Wistar rats, 180 - 220 g) used in this study were purchased from the Institute Pasteur (Tehran, Iran) and kept under standard temperature and relative humidity conditions. Before the experiments, the rats were habituated for 1 week. All experiments were conducted following the standards and protocols of the ethics committee of Shahid Beheshti University of Medical Sciences in Tehran, Iran (ID: IR.SBMU.PHARMACY.REC.1401.087). Renal proximal tubular cells (RPTCs) were isolated from Wistar rats (29, 30).

### 3.2. Renal Proximal Tubular Cells Isolation

Renal proximal tubular cells were isolated using the method described by Boom et al., and Schafer et al. (29, 30). Before digesting the samples, the kidneys were perfused with Hank's balanced salt solution (HBSS), which is free of Ca^2+^. The next step involved using collagenase type II HBSS, which contains penicillin/streptomycin and calcium chloride. De-capsulation and subsequent dissection of renal cortical segments were performed to generate 0.5 mm-thick sections and proximal cell tubules, which were mechanically separated based on serial filtration (120 μm and 60 μm meshes). Renal proximal tubular cells were then washed and pelleted before being re-suspended in Earle's solution (pH = 7.4) as an incubation medium. This step was carried out in a round vessel with a round bottom that circulated in a water bath. Finally, 28 mM HEPES was added to the incubation medium and covered with O_2_, N_2_, CO_2_, 10%, 85%, and 5%, respectively (31).

### 3.3. Cytotoxicity Assay

An indicator of cytotoxicity was lactate dehydrogenase (LDH) released by cells. Specifically, LDH activity was measured using an LDH kit from Sigma-Aldrich Co. 10 µL of sample and indicator (1 mL) were mixed at 37°C. Additionally, associated absorption was measured at 30-second intervals over a 4-minute period. A relative coefficient averaged the absorbance of the samples at 340 nm (Beckman DU-7 spectrophotometer) into units of enzyme activity. Lactate dehydrogenase activity for each treatment group was calculated as µM (substrate)/min/L (32).

### 3.4. Mitochondrial Isolation

**Figure 1.**
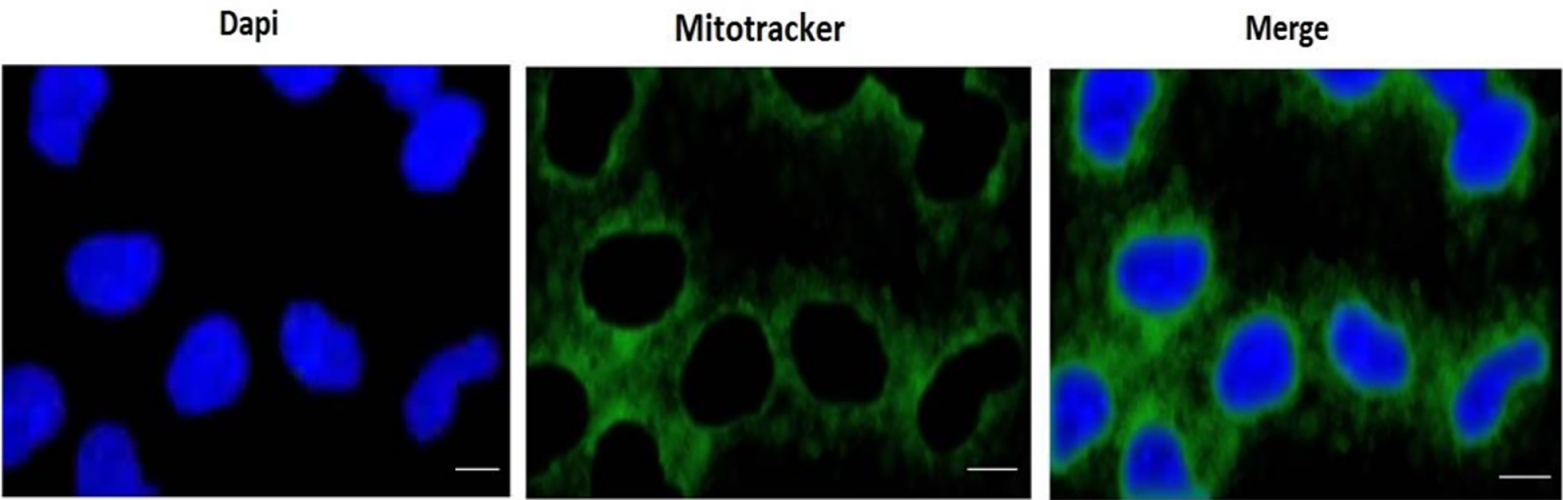
Mitochondrial bio-distribution. Internalization of mitochondria (green labeled) into renal proximal tubular cells (RPTCs) during 4 h of incubation (scale bar: 50 µm)

In recent studies, active fresh mitochondria were prepared from Wistar rat kidneys using the process of differential ultracentrifugation (Hettich, Universal 320 R, Germany) (33, 34). Rat kidneys were removed in a cold isolation solution and minced with scissors. A homogenization procedure was performed using a glass homogenizer, followed by the removal of intact cells and nuclei through centrifugation for 10 minutes at 4°C. After adding 250 μL of BSA solution to the supernatant, filtration was conducted with a mesh size of 4 or 5 μm. Additionally, the supernatant was centrifuged at 10 000 × g for 10 minutes. The bottom layer, known as the mitochondrial fraction, was re-suspended in an isolation solution. It underwent centrifugation twice for 10 minutes at 10 000 × g. The mitochondrial fraction was suspended using an incubation buffer (Tris buffer) at 4°C (pH 7.4). The bio-distribution of mitochondria is illustrated in Figure 1. 

### 3.5. Fluorescence Microscopy Image Capturing, Staining of the Nuclei of RPTCs by DAPI and Mitochondrial Staining by Mito-Tracker Green

A 1 mL sample of untreated control RPTCs was washed with PBS and immobilized with a paraformaldehyde PBS solution (3.7%) at room temperature for 10 minutes. The immobilized cells were then washed with PBS and stained with DAPI. In the next step, the RPTCs were washed again with PBS and observed using a fluorescence microscope. Under the fluorescence microscope, the fluorescence of the cells in the control group appeared more diffuse and uniform, the nucleus was relatively complete and regular, with an ellipsoid shape, and the nucleolus was clearly visible (Figure 1). 

The isolated mitochondria were suspended in Tris-HCl buffer and stained with Biotracker (mitotracker) 488 green. Subsequently, the mitochondria were observed using a fluorescence microscope. Under the fluorescence microscope, the mitochondria appeared as short rods or ball-shaped structures, with regular shapes (Figure 1). 

### 3.6. Experiment Design

First, Rat RPTCs were isolated and suspended in Earle's solution (pH 7.4). Then, the Rat RPTCs were incubated with DOX (5 µM) for 2 hours. Next, to study the effects of mitochondrial transplantation on the effects caused by DOX, fresh mitochondria were isolated from rat kidney. The RPTCs medium was replaced. Subsequently, control RPTCs as well as RPTCs exposed to DOX were incubated with freshly isolated mitochondria for 4 hours in a 37°C water bath. After the completion of incubation, the parameters/tests were performed (31).

### 3.7. Reactive Oxygen Species Determination

Using DCFH-DA reagent and fluorescence intensity measurement, we evaluated the ROS level. Mitochondrial membrane potential (MMP) changes in groups treated with DOX (5 µM), mitochondrial transplantation (40 and 80 µg/mL), and cytochalasin D (10 µM) were measured by DCFH-DA reagent and a spectrophotometer at wavelengths of 500 nm and 520 nm (35).

### 3.8. MMP Assay

Using rhodamine 123 (Rh123) reagent and fluorescence intensity measurement, we evaluated the collapse in the MMP. Mitochondrial membrane potential changes in groups treated with DOX (5 µM), mitochondrial transplantation (40 and 80 µg/mL), and cytochalasin D (10 µM) were measured by Rh123 reagent using a spectrophotometer at wavelengths of 490 nm and 520 nm (36).

### 3.9. GSH and GGSG Content Assay

In this study, we evaluated the GSH and GSSG content in groups treated with DOX (5 µM), mitochondrial transplantation (40 and 80 µg/mL), and cytochalasin D (10 µM) using a spectrophotometer at wavelengths of 350 nm and 420 nm (37).

### 3.10. Evaluation of Lipid Peroxidation

The content of malondialdehyde (MDA) was evaluated to measure lipid peroxidation (LPO) as one of the parameters indicating oxidative stress. Trichloroacetic acid (TCA, 10%) and thiobarbituric acid (TBA, 0.37%) were used in the assay. The amounts of the compounds used were as follows: TCA 750 µL, TBA 500 µL, and 250 µL supernatant. After centrifugation at 10 000 × g for 10 min, 250 µL of the samples were used for the assay. Finally, the content of MDA was assayed using a spectrophotometer at a wavelength of 532 nm (38).

### 3.11. Caspase-3 Activity Measurement

The caspase-3 kit (Sigma-Aldrich) was used to measure caspase-3 according to the manufacturer's instructions (39).

### 3.12. Mitochondrial Uptake Mechanism Evaluation

The mitochondrial uptake mechanism was evaluated by pre-treatment with 5-(N-ethyl-N-isopropyl) amiloride (EIPA) (100 µM) (40), cytochalasin D (10 µM) (41), and methyl-β-cyclodextrin (1 mM) (42) in DOX-treated RPTCs (10^6^ cells/mL). The aforementioned pre-treatments were conducted for 30 minutes in three separate flasks to compare the results with those of RPTCs treated only with DOX (10^6^ cells/mL) in the fourth flask. Isolated mitochondria (80 μg protein/mL) were then added to each flask and co-incubated at 37°C with 5% CO_2_ for 4 hours. Finally, the ATP levels in the four flasks were assessed (43).

### 3.13. Statistical Evaluation

Data representation processing was performed as mean ± SD. The data were analyzed using GraphPad Prism 6 (GraphPad, La Jolla, CA, USA). Additionally, P < 0.05 was chosen as the minimum significance level. The one-way ANOVA test followed by the post hoc Tukey test was used for the evaluation of all tests.

## 4. Results

### 4.1. Mitochondrial Transplantation Reduced DOX Induced Cytotoxicity

**Figure 2.**
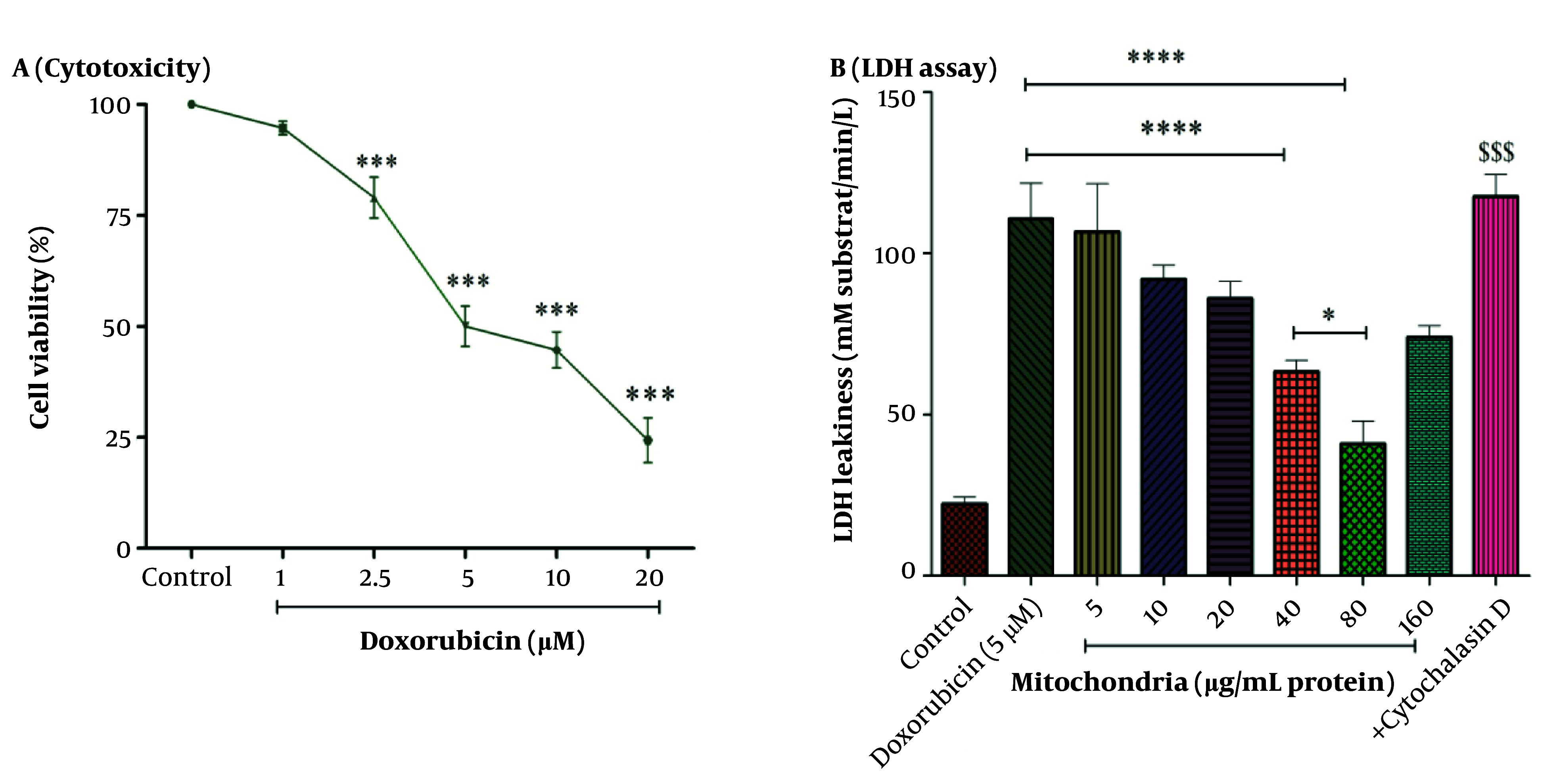
A, cytotoxicity assay; and B, LDH assay. DOX at concentrations of 2.5 to 20 µM has decreased cell viability in RPTCs. IC_50_ DOX was 5 µM. Mitochondrial transplantation (40 and 80 µg/mL protein) was able to reduce DOX-induced cytotoxicity. Values were represented as mean ± SD (n = 5). *** P < 0.001 vs control group; **** P < 0.0001 vs DOX group; * P < 0.05 vs mitochondria group (40 µg/mL); $$$ P < 0.001 vs mitochondria group (40 and 80 µg/mL). DOX, doxorubicin; LDH, lactate dehydrogenase; RPTCs, renal proximal tubular cells.

The results in Figure 2 showed that DOX caused toxicity in RPTCs at concentrations ranging from 1 to 20 µM (1, 2.5, 5, 10, and 20 µM) in a concentration-dependent pattern. Also, the concentration of 5 µM has been reported as an IC_50_ concentration (Figure 2A). A concentration of 5 µM was used for the experiments. To determine the best protective concentration for the isolated kidney mitochondria used for mitochondrial transplantation in reducing the toxicity of DOX (5 μM) in RPTCs, we transplanted several concentrations of isolated kidney mitochondria (5 - 160 μg protein/mL) onto DOX-treated damaged RPTCs. The results showed that transplantation of mitochondria at concentrations of 40 and 80 µg/mL protein significantly protected the DOX-treated rat RPT cells against the cytotoxicity caused by DOX (5 µM) compared to other concentrations of isolated kidney mitochondria (Figure 2B). Consequently, the concentrations of 40 and 80 µg/mL protein of mitochondria were used to investigate the effects of mitochondrial transplantation.

### 4.2. Mitochondrial Transplantation Decreased DOX Induced Reactive Oxygen Species Formation

**Figure 3.**
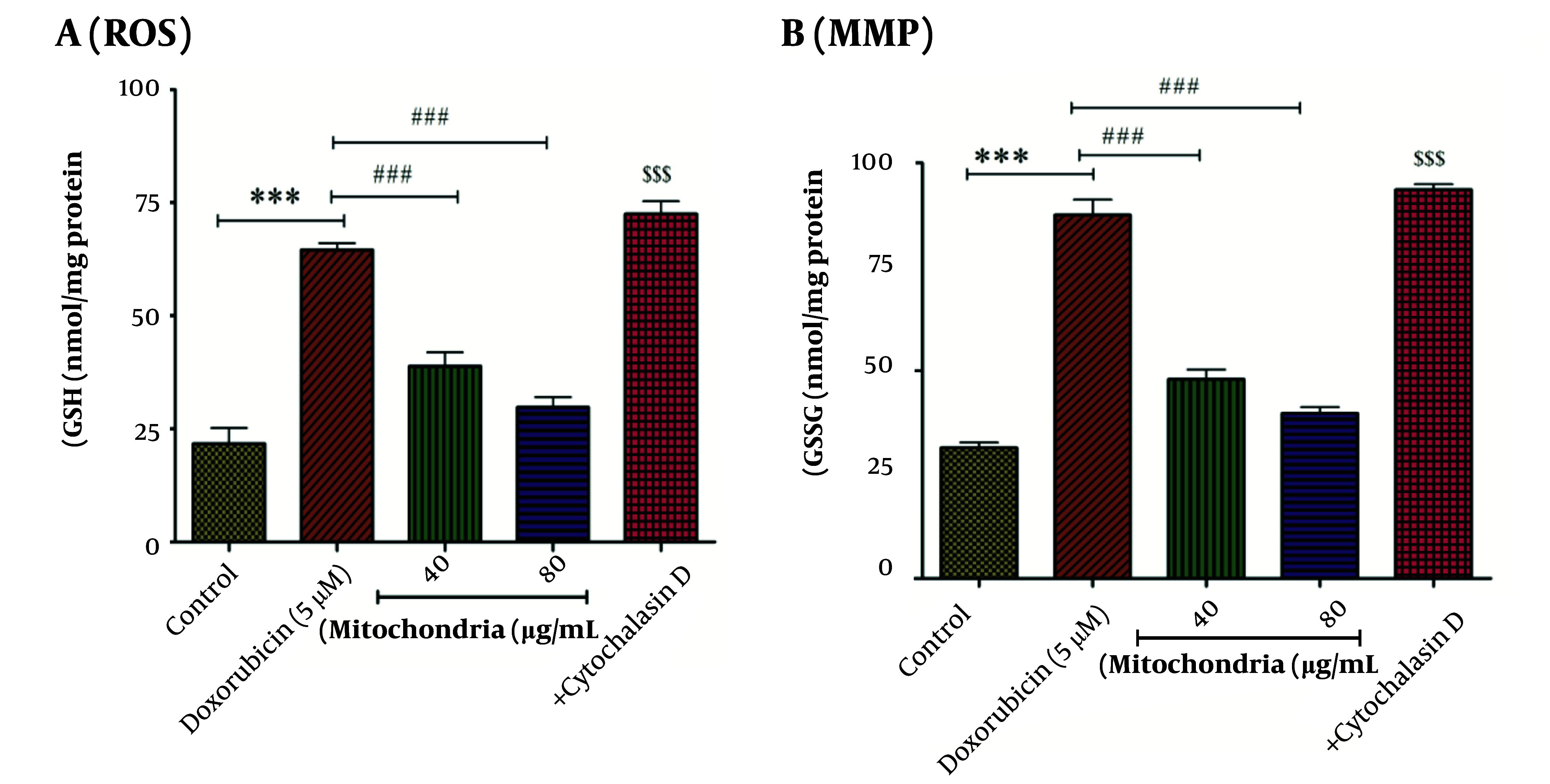
A, ROS assay. DOX (5 µM) significantly increased the level of ROS in RPTCs. Mitochondrial transplantation (40 and 80 µg/mL protein) has improved the effect (ROS generation) caused by DOX (5 µM). Values were represented as mean ± SD (n = 5). *** P < 0.001 vs control group; ### P < 0.001 vs DOX group; $$$ P < 0.001 vs mitochondria group (40 and 80 µg/mL). B, MMP assay. There is a direct correlation between fluorescence intensity and collapse in MMP. Mitochondrial transplantation (40 and 80 µg/mL protein) has been able to significantly reduce the effects of DOX (5 µM) on changes in the mitochondrial membrane of RPTCs. Values were displayed as mean ± SD (n = 5). *** P < 0.001 vs control group; ### P < 0.001 vs DOX group; $$$ P < 0.001 vs mitochondria group (40 and 80 µg/mL). ROS, reactive oxygen species; DOX, doxorubicin; RPTCs, renal proximal tubular cells; MMP, mitochondrial membrane potential.

Our results indicated that DOX (5 µM) induces the production of ROS in RPTCs. As seen in Figure 3A, this approach (40 and 80 µg/mL protein of mitochondria) has reduced the effect of DOX (5 µM) on the production of ROS in RPTCs (Figure 3A). This inhibitory effect of this therapeutic approach can help reduce the damage caused by DOX in RPTCs. Cytochalasin D causes an increase in the level of ROS, which is similar to DOX (5 µM) (Figure 3A). 

### 4.3. Mitochondrial Transplantation Decreased DOX Induced MMP Collapse

After 2 hours of incubation and compared to the control group, DOX (5 µM) has caused a disturbance in the MMP (Figure 3B). 40 and 80 µg/mL protein of mitochondria were used to investigate the beneficial effects of this therapeutic approach. After 4 hours of incubation, we indicated that the mitochondrial transplantation (40 and 80 µg/mL) reduced the effects of DOX (5 µM) on MMP changes. The effects of cytochalasin D on MMP are similar to DOX (5 µM) (Figure 3B). Mitochondrial membrane potential is involved in the health and death of mitochondria.

### 4.4. Mitochondrial Transplantation Improved DOX Decreased GSH Content

**Figure 4.**
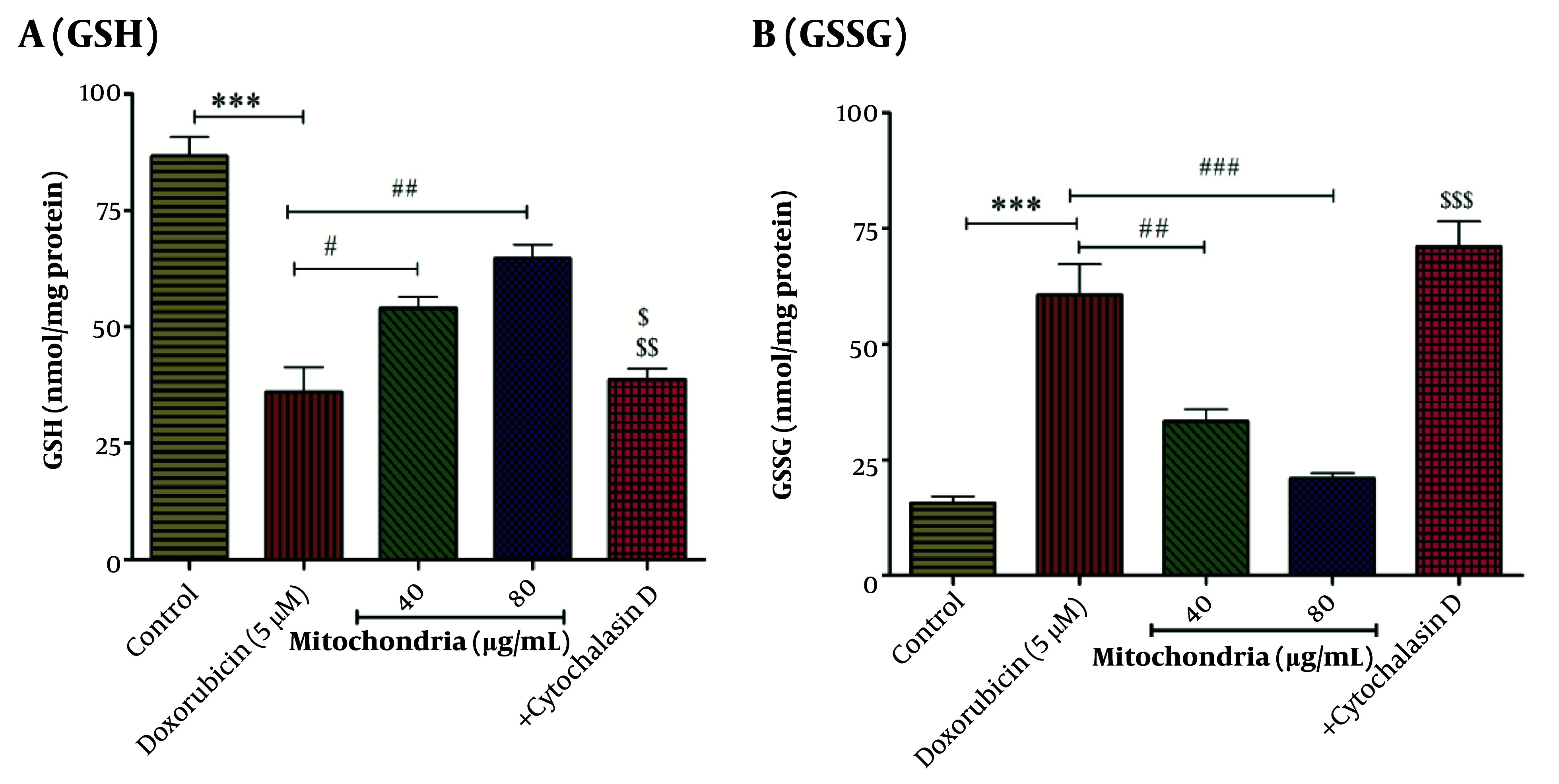
A, GSH assay; B, GSSG assay. Mitochondrial transplantation (40 and 80 µg/mL protein) was able to significantly increase DOX (5 µM)-induced reduced GSH levels and also reduce DOX (5 µM)-induced increased GSSG levels. Values were displayed as mean ± SD (n = 5). *** P < 0.001 vs control group; # P < 0.05, ## P < 0.01 and ### P < 0.001 vs DOX group; $ P < 0.05 vs mitochondria group (40 µg/mL); $$ P < 0.01 vs mitochondria group (80 µg/mL); $$$ P < 0.001 vs mitochondria group (40 and 80 µg/mL). DOX, doxorubicin; GSH, reduced glutathione; GSSG, oxidized glutathione.

**Figure 5.**
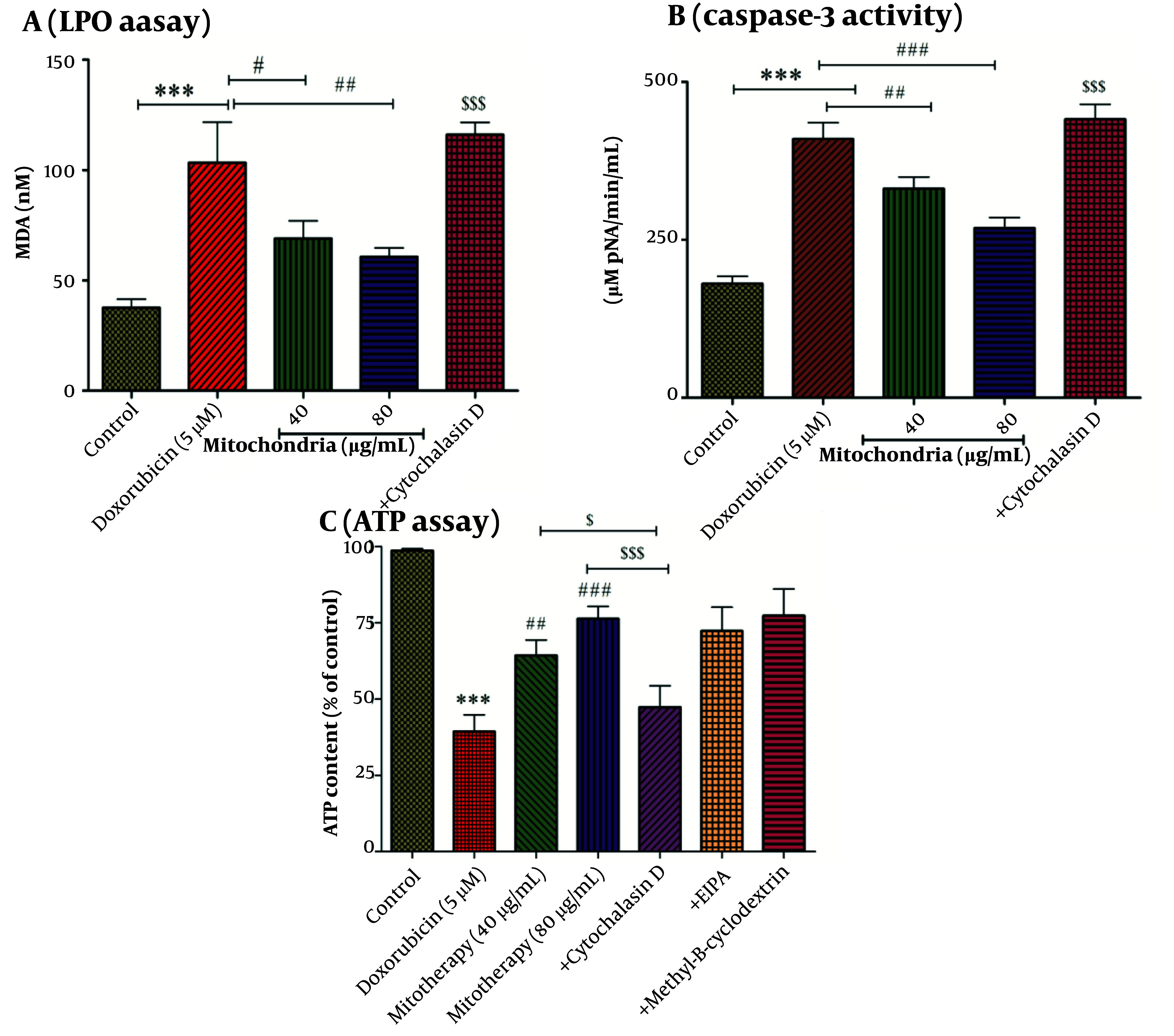
A, LPO assay. LPO is one of the important indicators of oxidative stress. Mitochondrial transplantation (40 and 80 µg/mL protein) was able to significantly decrease DOX (5 µM)-induced LPO levels. Values were displayed as mean ± SD (n = 5). *** P < 0.001 vs control group; # P < 0.05 and ## P < 0.01 vs DOX group; $$$ P < 0.001 vs mitochondria group (40 and 80 µg/mL). B, caspase-3 activity assay. Mitochondrial transplantation (40 and 80 µg/mL protein) caused a decrease in caspase-3 activity, which was increased by DOX (5 µM) in RPTCs. Values were displayed as mean ± SD (n = 5). *** P < 0.001 vs control group; ## P < 0.01 and ### P < 0.001 vs DOX group; $$$ P < 0.001 vs mitochondria group (40 and 80 µg/mL). C, ATP assay. After 2 hours of incubation, DOX (5 µM) caused a decrease in the ATP level. Mitochondrial transplantation (40 and 80 µg/mL protein) ameliorated this DOX (5 µM)-induced effect (reduction in ATP levels). Values were displayed as mean ± SD (n = 5). *** P < 0.001 vs control group; ## P < 0.01 and ### P < 0.001 vs DOX group; $ P < 0.05 vs mitochondria group (40 µg/mL); $$$ P < 0.001 vs mitochondria group (80 µg/mL). DOX, doxorubicin; LPO, lipid peroxidation, RPTCs, renal proximal tubular cells; ATP, adenosine triphosphate.

Based on statistical analysis, the results revealed that DOX (5 µM) decreased the content of GSH in RPTCs (Figure 4A). As seen in Figure 5, mitochondrial transplantation (40 and 80 µg/mL protein of mitochondria) has been able to increase the GSH content decreased by DOX (5 µM) in RPTCs (Figure 4A). Also, the results showed that mitochondrial transplantation caused the reduction of GSSG content induced by DOX (5 µM) in RPTCs (Figure 4B). The results showed that cytochalasin D increases the GSSG level and decreases the GSH level (Figure 4A-B). This reduction effect of this therapeutic approach shows the improvement of antioxidant status in RPTCs.

### 4.5. Mitochondrial Transplantation Decreased DOX Induced LPO

Compared to the control group, DOX has been able to increase the content of MDA as an indicator of LPO (Figure 5A). After 4 hours of incubation, the results showed that the mitochondrial transplantation (40 and 80 µg/mL) was able to reduce the effects of DOX (5 µM) on MDA content (Figure 5A). As shown in Figure 5A, cytochalasin D causes an increase in the content of LPO.

### 4.6. Mitochondrial Transplantation Decreased DOX Induced Caspase-3 Activity

Incubation of RPTCs with DOX at a concentration of 5 µM has been associated with caspase-3 activation in RPTCs (Figure 5B). Furthermore, we indicated that mitochondrial transplantation can reduce the activity of caspase-3, which is increased by DOX (5 µM) (Figure 5B). Therefore, mitochondrial transplantation with this effect can prevent cell death caused by DOX.

### 4.7. Cytochalasin D Reduced the Restoration Effect of Mitochondrial Transplantation on ATP Content

Based on statistical analysis, the results revealed that DOX (5 µM) was able to decrease the content of ATP in RPTCs (Figure 5C). As seen in Figure 5C, mitochondrial transplantation (40 and 80 µg/mL protein of mitochondria) has been able to increase the ATP content decreased by DOX (5 µM) in RPTCs. We reported that cytochalasin D-based pre-incubation may considerably inhibit mitochondrial transplantation protective effects. We indicated that only cytochalasin D affected ATP content in RPTCs. Furthermore, the mechanistic results showed that EIPA (macropinocytosis inhibitor/100 µM) and methyl-β- Cyclodextrin (caveola/clathrin-dependent endocytosis inhibitor/1 mM) as two inhibitors could not reverse the effects caused by mitochondrial transplantation. Accordingly, these two inhibitors did not play a role in mitochondria internalization (Figure 5C). As a result, the actin-dependent endocytosis would play a role in the mitochondria's internalization into the RPTCs (Figure 5C). 

### 4.8. Fluorescence Microscopy for Showing Mitochondrial Uptake by the DOX Treated RPTCs

To demonstrate mitochondrial uptake by the DOX-treated RPTCs, the incubation media of DOX-treated RPTCs were replaced with kidney mitochondria stained with mitotracker green dispersed in the same incubation medium (Earle's solution, pH = 7.4) at a concentration of 80 µg/mL mitochondrial protein, which had already shown the best protective effect against DOX cytotoxicity. The mitochondrial transplantation took place 2 hours after DOX addition to the RPRCs. Then, 4 hours later, 7 toxicity parameters including cell lysis, ROS formation, MMP decline, GSH and GSSG content, LPO, ATP content, and finally Caspase-3 activity (the final mediator of apoptosis) were measured to determine the probable protective effect of mitochondrial transplantation (Figure 1). 

## 5. Discussion

DOX is an anti-cancer drug used to treat various cancers. However, its use is limited due to its effects on different organs as well as healthy cells (44, 45). It has been reported that DOX can affect mitochondria and disrupt their function (19, 20, 46). The kidney is one of the organs with a high number of mitochondria due to its function. Additionally, there is a direct link between normal mitochondrial function and mitochondrial health (47). Renal proximal tubules are one of the parts that DOX can affect. Also, this anti-cancer drug induces cell death signaling through the mitochondrial pathway in this part of the kidney (21, 22). The number of mitochondria in renal tubules is high for ATP production. Therefore, the effect on mitochondria can be associated with many disorders in tubules (47).

Mitochondria are involved in many physiological processes, the most important of which include ATP production, free radicals production, differentiation, and apoptosis. Mitochondria are considered the most important source of energy (ATP) for kidneys. Tubular defects are among the most common kidney symptoms caused by mitochondrial dysfunction. Disruption in the normal function of mitochondria is recognized as one of the significant causes of several diseases (31). Therefore, it is necessary to explore new therapeutic approaches to treat mitochondria-related disorders. Nowadays, researchers have been investigating the use of mitochondrial transplantation as a new treatment approach for mitochondrial disorders. In this approach, healthy exogenous mitochondria are transferred to damaged cells (48-50).

The internalization of exogenous mitochondria into RPTCs was confirmed in our research. Furthermore, our study demonstrated that mitochondrial transplantation reduced DOX-induced cytotoxicity in RPTCs. In this study, the internalization of mitochondria into the RPTCs was observed using fluorescence microscopy techniques with DAPI and Mito-Tracker Green double staining. However, other confirmatory tests, such as flow cytometry for the quantitative determination of the uptake of isolated kidney mitochondria into RPTCs, were not performed, which could be considered one of the limitations of the study. Previous studies have indicated that DOX causes nephrotoxicity through oxidative stress (5, 45, 51). Oxidative stress is a condition in which the level of oxidant factors, especially free radicals, is higher than the level of antioxidant factors (16). In kidney tubules, the mitochondrial electron transport chain is recognized as the primary source of ROS production (47). It has been reported that DOX induces kidney toxicity through its effect on mitochondrial electron transport, which can lead to ROS production (46). Our findings revealed that DOX induces the production of ROS in kidney cells, consistent with previous studies (5, 14, 15). In this study, mitochondrial transplantation reduced the production of ROS caused by DOX in RPTCs. Therefore, this approach may be effective in preventing oxidative stress, which is considered one of the important mechanisms in causing nephrotoxicity induced by DOX.

Mitochondrial membrane potential plays a crucial role in mitochondrial function, and its disruption is associated with damage to mitochondria and other consequences, such as mitochondrial swelling and induction of cell death (18). Mitochondrial transplantation was able to reduce the collapse in MMP induced by DOX in RPTCs. Based on this, mitochondrial transplantation with this effect can prevent other events, such as the release of pro-apoptotic proteins that cause cell death. Previous studies have shown that DOX reduces the activity of the antioxidant system by reducing GSH activity, providing evidence of oxidative stress (5, 14, 52). GSH is involved in the detoxification of xenobiotic agents and the removal of ROS. One of the pieces of evidence of oxidative stress is a result of the decrease in GSH levels (53). In agreement with our previous studies, our results showed that DOX has reduced GSH levels in RPTCs (4, 5). Also, mitochondrial transplantation has been able to improve the decreased GSH levels caused by DOX. Another important indicator of oxidative stress is the level of LPO, which is reported by measuring the content of MDA. Similar to other studies, our report showed that DOX can cause LPO (10, 45). Mitochondrial transplantation also can reduce LPO caused by DOX. These results are in agreement with previous studies that have shown that mitochondrial transplantation reduces LPO (54, 55). Therefore, mitochondrial transplantation can reduce the level of oxidative stress, which is one of the most important mechanisms involved in DOX toxicity.

There is a direct relationship between oxidative stress and cell vulnerability. It can also play a role in the initiation of apoptosis. Caspase-3 is one of the important factors involved in apoptosis signaling. It has been shown that caspase-3 level is involved in nephrotoxicity (56). Our results showed that this treatment approach was able to reduce caspase-3 activity in RPTCs, which can prevent cell death with this effect. This reported effect of mitochondrial transplantation aligns with past studies (57). Renal tubular cells need ATP to perform their function. Therefore, a decrease in its level causes damage to its performance. Mitochondrial transplantation has been able to increase ATP content in RPTCs. This effect is in agreement with previous studies (58).

### 5.1. Conclusions

In our study, we proposed a therapeutic strategy involving the transplantation of exogenous mitochondria for the treatment of kidney impairment. Our results suggest that mitochondrial transplantation decreases DOX-induced cytotoxicity in rat RPTCs. Furthermore, this therapeutic strategy reduces oxidative stress and damage to mitochondria caused by DOX in rat RPTCs.

## Data Availability

The dataset presented in the study is available on request from the corresponding author during submission or after publication.
